# Index Medicus

**Published:** 1879-01

**Authors:** 


### Index Medicus.

A monthly classified record of the current medical literature of the world. Compiled under the supervision of Dr. John S. Billings, Surgeon U. S. Army, and Dr. Robert Fletcher, M.R.C.S. Eng. F. Leypoldt, publisher, 37 Park Row, New York.

The *Index Medicus* will record the titles of all new publications in Medicine, Surgery, and the collateral branches, received during the preceding month. These will be classed under subject headings, and will be followed by the titles of valuable original articles upon the same subject, found during the like period, in medical journals and transactions of medical societies. The periodicals thus indexed will comprise all current medical journals and transactions of value so far as they can be obtained. At the close of each yearly volume a double index of authors and subjects will be added, forming a complete bibliography of medicine for the preceding year.

The first number of the *Index* will appear in January, 1879.

It is not at present intended to index journals devoted to subjects of Chemistry, Pharmacy, Veterinary Medicine, and Dentistry, but the editors will select from them articles which have a bearing upon pathology and therapeutics.

In the description of new books *received*, the size, price, illustrations, place of publication, and publisher's name will be accurately given. Publishers are requested to send advance copies of their works to insure an early insertion in the *Index.*

Few words are required to demonstrate the utility of the projected serial. In its pages the practitioner will find the titles of parallels for his anomalous cases, accounts of new remedies, and the latest methods in therapeutics.

The teacher will observe what is being written or taught by the masters of his art in all countries. The author will be enabled to add the latest views and cases to his forthcoming work, or to discover where he has been anticipated by other writers, and the publishers of medical books and periodicals must necessarily profit by the publicity given to their publications.

Books, journals, and communications for the editors, from parties resident in America, should be addressed to Editors of the Index Medicus, Washington, D. C.

The *Index Medicus* will be published monthly, to range with the leading medical journals (which it supplements as a current guide to all), at No. 37 Park Row, New York. The subscription price will be $3 per year, payable in advance. Prompt subscriptions in support of the enterprise are respectfully solicited.

